# Hemoperitoneum, Hepatic Laceration, and Hepatic Artery Pseudoaneurysm as a Complication of Emergent Pericardiocentesis

**DOI:** 10.1016/j.jaccas.2022.101686

**Published:** 2022-11-29

**Authors:** Waseem Farooq, Vijay Iyer

**Affiliations:** University at Buffalo, State University of New York, Buffalo, New York, USA

**Keywords:** hemoperitoneum, hepatic artery laceration, pericardiocentesis, pericardial tamponade, CTA, computed tomography angiogram, LA, left atrium, MR, mitral regurgitation, TEE, transesophageal echocardiogram, TEER, transcatheter mitral valve edge-to-edge repair, TTE, transthoracic echocardiogram

## Abstract

Emergent pericardiocentesis is a potentially life-saving therapeutic procedure. We report a case of hemoperitoneum, a rare but known complication of pericardiocentesis; due to hepatic artery laceration and hepatic artery pseudoaneurysm formation resulting in delayed hemorrhagic shock as a complication of emergent pericardiocentesis. (**Level of Difficulty: Intermediate.**)

## Graphical abstract

**Figure:**
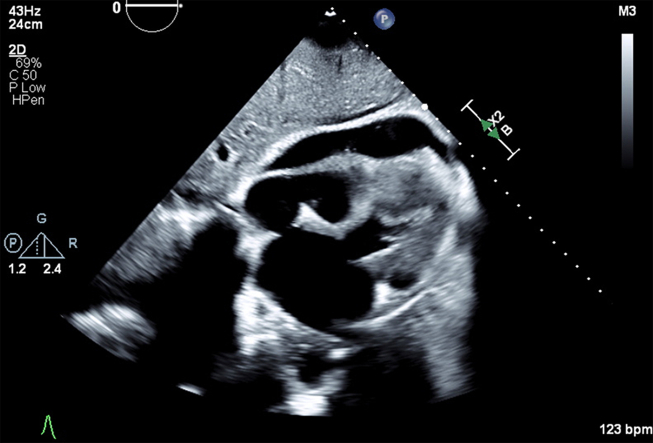

An 82-year-old woman with severe degenerative mitral regurgitation (MR) and NYHA functional class III symptoms, who was using maximally tolerated medical therapy, presented for elective transcatheter mitral valve edge-to-edge repair (TEER).

**Learning Objectives**

• To describe a rare complication of pericardiocentesis including hemoperitoneum secondary to hepatic laceration and hepatic artery pseudoaneurysm formation.
• To discuss management of hepatic artery laceration and pseudoaneurysm.
• To recognize unforeseen complications resulting from prolonged hospital stay.

The patient underwent successful TEER with a MitraClip device by the use of fluoroscopy and transesophageal echocardiography (TEE). After the procedure, good hemostasis was accomplished. and TEE did not reveal any pericardial effusion. The patient was extubated and transferred to the recovery room in stable condition.

Ninety minutes after the procedure, the patient became hypotensive; her mean arterial pressure (MAP) was 37 mm Hg, and she was tachycardic (heart rate 144 beats/min), and encephalopathic. A rapid response was called, and a bedside transthoracic echocardiogram (TTE) revealed new-onset moderate, predominantly anterior, pericardial effusion with pericardial tamponade (Figure 1, Video 1).

**Figure 1:**
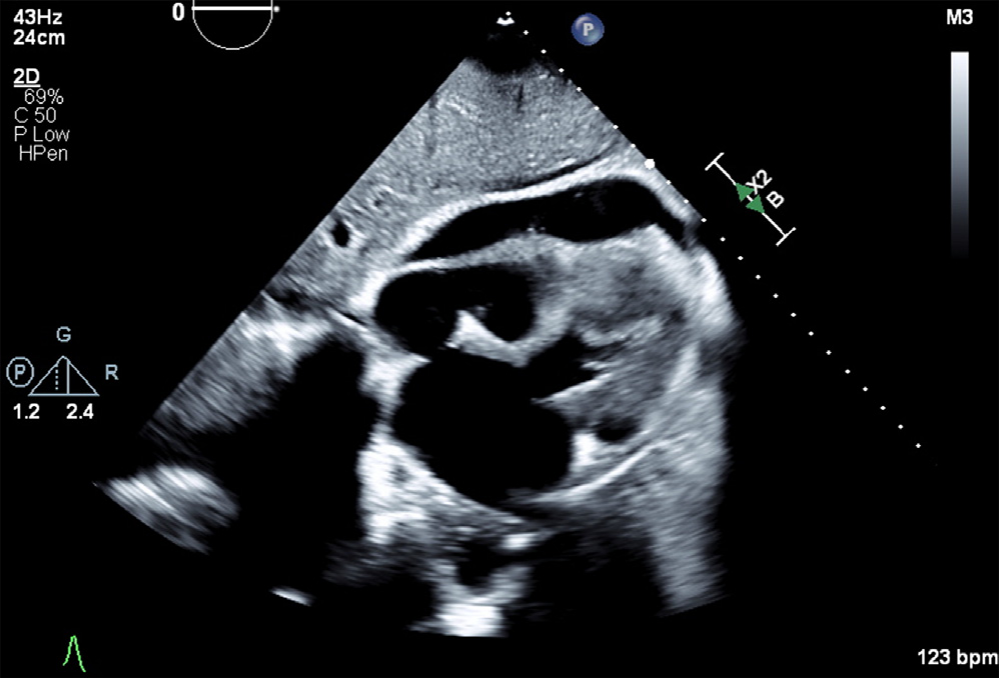
Transthoracic Echocardiogram Subcostal view with new-onset moderate, predominantly anterior, pericardial effusion.

The patient was resuscitated with intravenous (IV) fluids and vasopressors for hemodynamic support. No chest compressions were done. Bedside attempts to perform pericardiocentesis by a substernal approach using ultrasound guidance were unsuccessful because of her inability to lie flat and her small body frame. The patient was taken to the catheterization laboratory and underwent successful pericardiocentesis through a fluoroscopy-guided substernal approach, with removal of 270 mL bloody fluid and no significant residual effusion on TTE after the procedure. An 8-F pigtail catheter was placed.

On day 2, the patient reported right lower quadrant abdominal pain. TTE revealed no significant pericardial effusion (Figure 2, Video 2).

**Figure 2:**
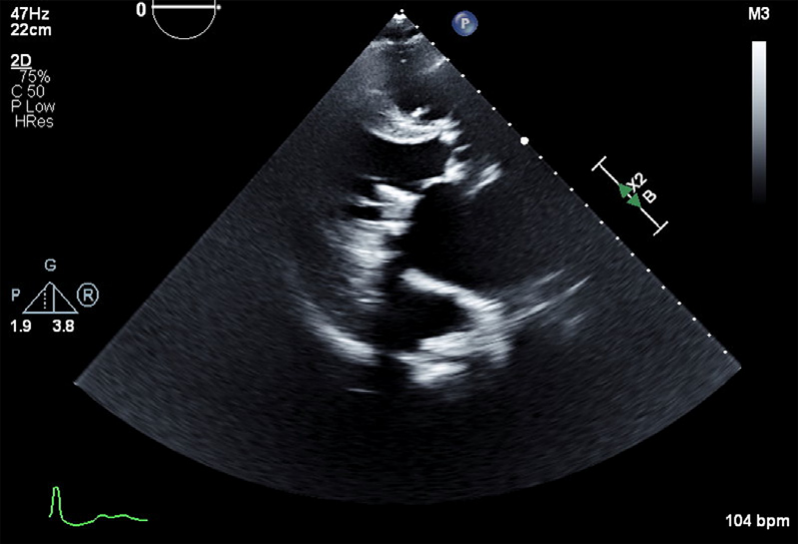
Transthoracic Echocardiogram Parasternal long-axis view showing resolution of pericardial effusion after pericardiocentesis and pigtail catheter placement.

After the procedure, the patient's hemoglobin dropped from 13.4 g/dL to 7.9 g/dL prompting a packed red blood cell transfusion and computed tomography (CT) of the torso without IV contrast, which demonstrated hemoperitoneum (Figure 3).

**Figure 3:**
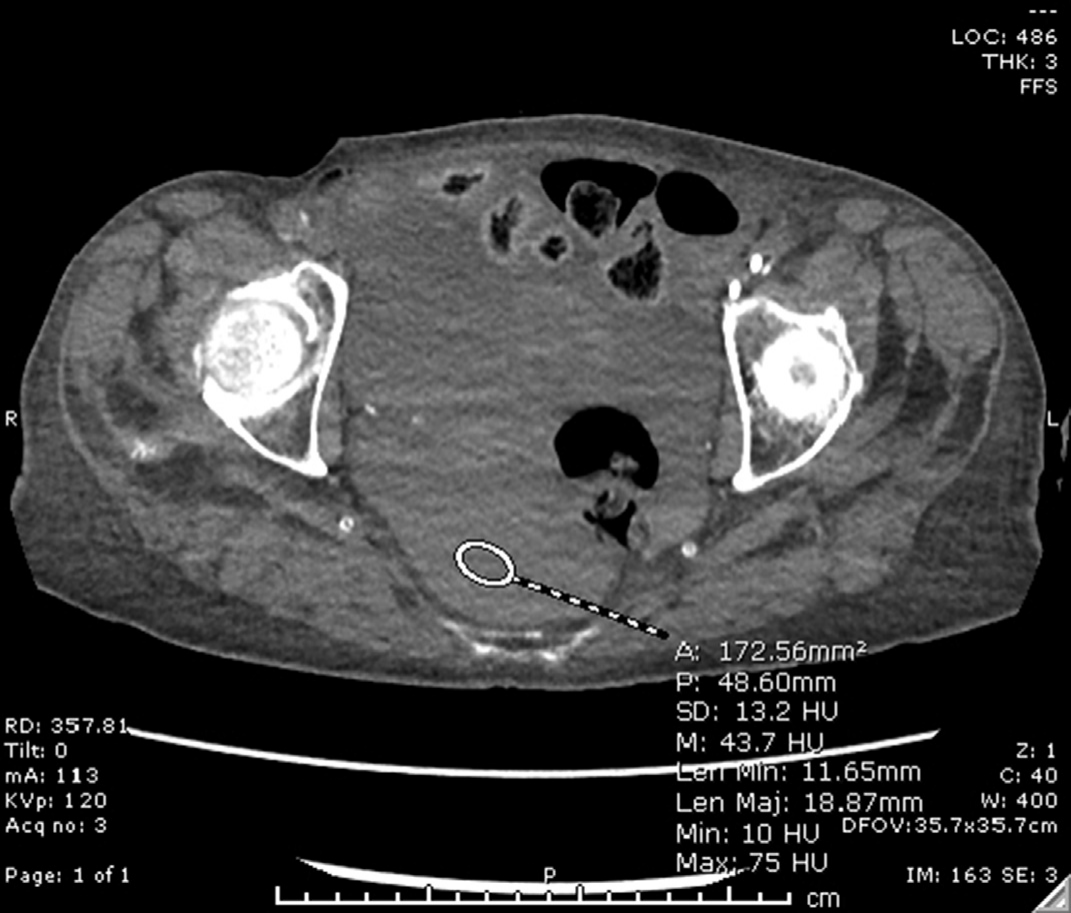
Noncontrast Computed Tomography of Abdomen View showing hemoperitoneum.

CT angiography (CTA) of the torso with IV contrast, obtained to evaluate the hemoperitoneum, revealed hyperdense fluid throughout the peritoneal cavity in keeping with hemoperitoneum and was stable from earlier noncontrast CT. An indeterminate hypodense lesion in the left lobe of the liver with surrounding hyperemia measuring 1.1 × 1.3 cm was also noted. Adjacent hyperenhancement on arterial phase represented some shunting. No evidence of active hemorrhage or blood pooling was noted. A pericardial drain was noted in situ without significant pericardial effusion (Figures 4 and 5).

**Figure 4:**
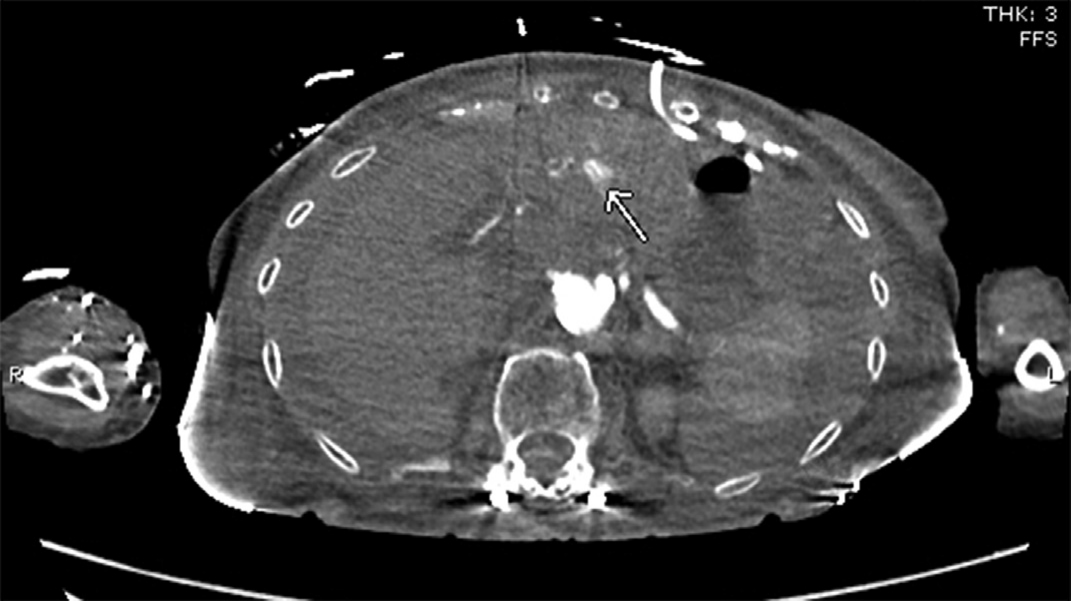
Computed Tomography Angiogram of Torso With Intravenous Contrast Material Hyperdense fluid throughout the peritoneal cavity in keeping with hemoperitoneum, stable from earlier noncontrast computed tomography. An indeterminate hypodense lesion **(arrow)** in left lobe of liver with surrounding hyperemia measuring 1.1 × 1.3 cm. Pericardial drain noted in situ.

**Figure 5:**
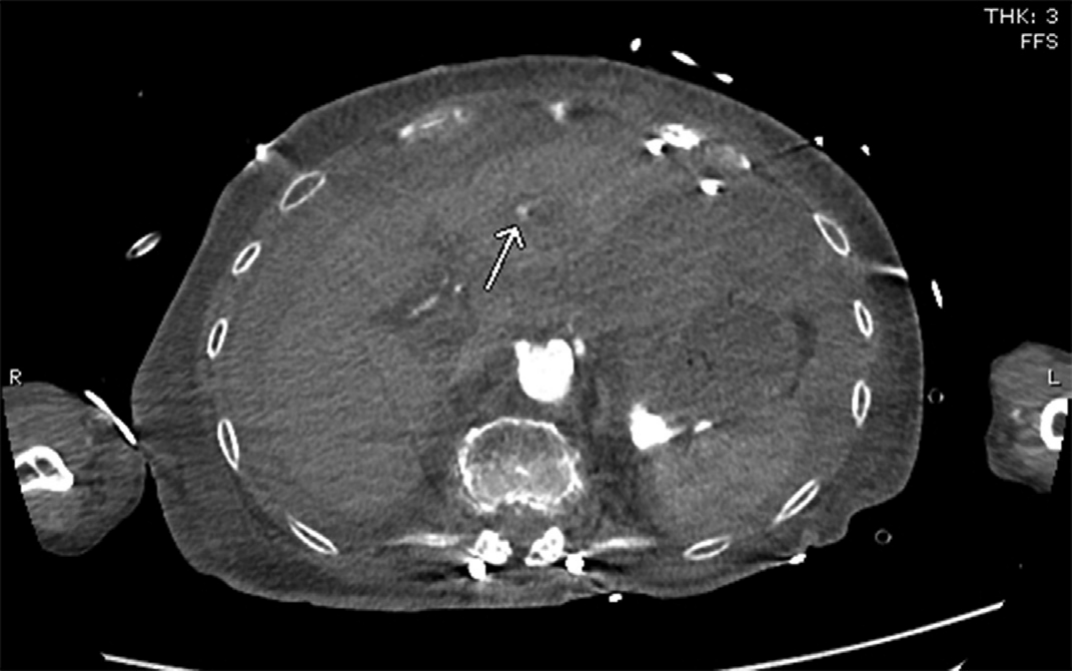
Computed Tomography Angiogram With Intravenous Contrast Material **Arrow** points to the adjacent hyperenhancement on arterial phase representing shunting.

The patient continued to require a relatively low dose of IV norepinephrine for vasopressor support. Continued TTE monitoring did not reveal any significant pericardial effusion, although the patient continued to have pericardial drain output.

On postprocedure day 3, the patient became unresponsive during placement of a peripherally inserted central catheter (PICC). Her MAP decreased to 40s mm Hg, with palpable pulses. IV norepinephrine was uptitrated. A bedside examination revealed soft but significantly distended abdomen.

## Medical History

The patient’s medical history included hypertension, right subclavian artery stenosis, carotid stenosis, coronary artery disease, atrial fibrillation, COPD, thyroid cancer, heart failure with mildly reduced EF at 45%, and MR.

## Differential Diagnosis

The differential diagnosis included pericardial effusion and hemoperitoneum secondary to pericardiocentesis.

## Investigations

**Figure 6:**
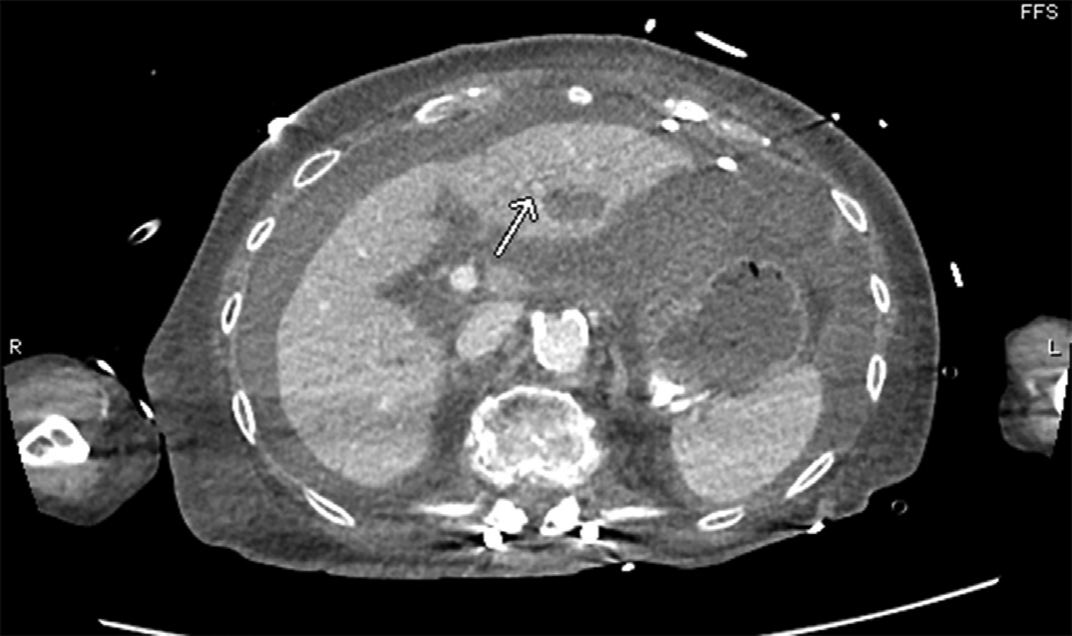
Computed Tomography Angiogram of Torso With Intravenous Contrast Material Enlarged hypoattenuating lesion within left lobe of liver, previously thought to be indeterminate mass, and now favored to represent liver laceration. **Arrow** points to ill-defined area of hyperattenuation on the arterial phase of the study without definite pooling on delayed phase representing intrahepatic extravasation and pseudoaneurysm formation.

Bedside TTE revealed unchanged nonsignificant pericardial effusion with no signs of hemodynamic compromise or tamponade. Bedside abdominal ultrasonography was concerning for free fluid/blood in the abdomen. Bedside i-STAT revealed a hematocrit of 15%. A complete blood count revealed hemoglobin of 5.1g/dL down from 7.5g/dL earlier that morning. After hemodynamic stabilization, the patient was immediately transported to the radiology suite for CTA of the torso, which revealed an enlarging hypoattenuating lesion within the left lobe of the liver, previously thought to be an indeterminate mass and now favored to represent liver laceration, with the pericardial drain lying in close proximity to it. Also noted were ill-defined areas of hyperattenuation on the arterial phase of the study without definite pooling on the delayed phase, representing intrahepatic extravasation and pseudoaneurysm formation. The hypoattenuating lesion/laceration now extended to the posterior capsule of the liver and was the source of moderate hemoperitoneum, which had increased slightly in volume from the prior CT (Figure 6).

## Management

**Figure 7:**
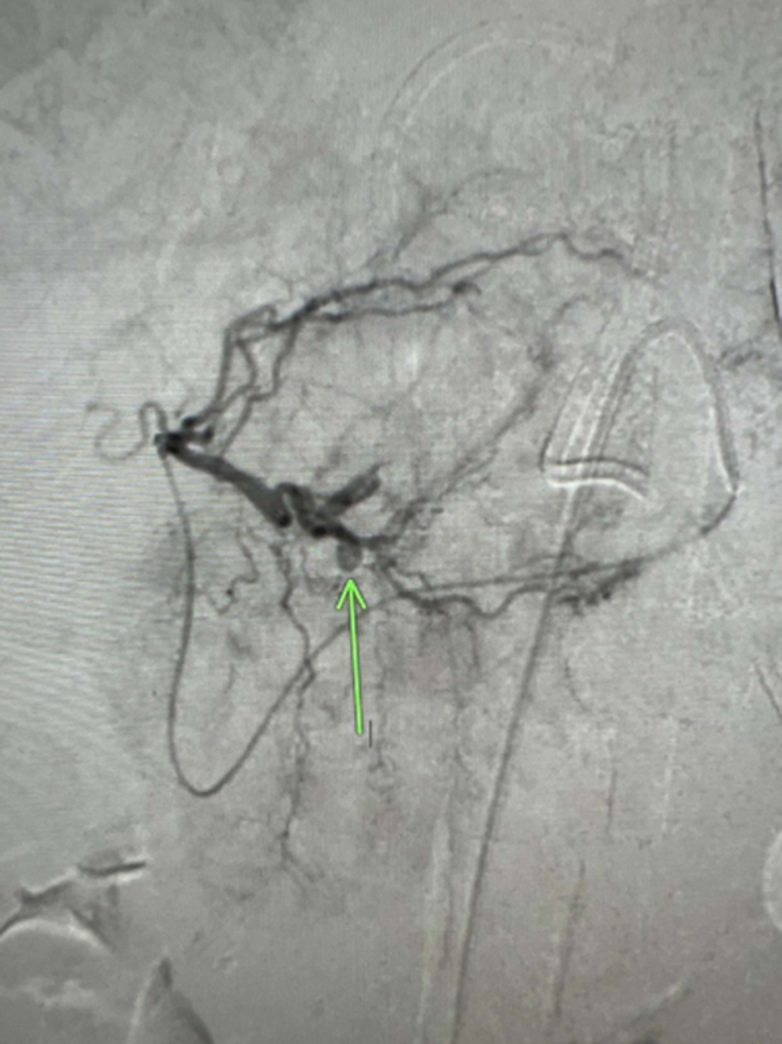
Digital Subtraction Angiography of Left Hepatic Artery Image demonstrating pseudoaneurysm arising from segment III branch of left hepatic artery.

**Figure 8:**
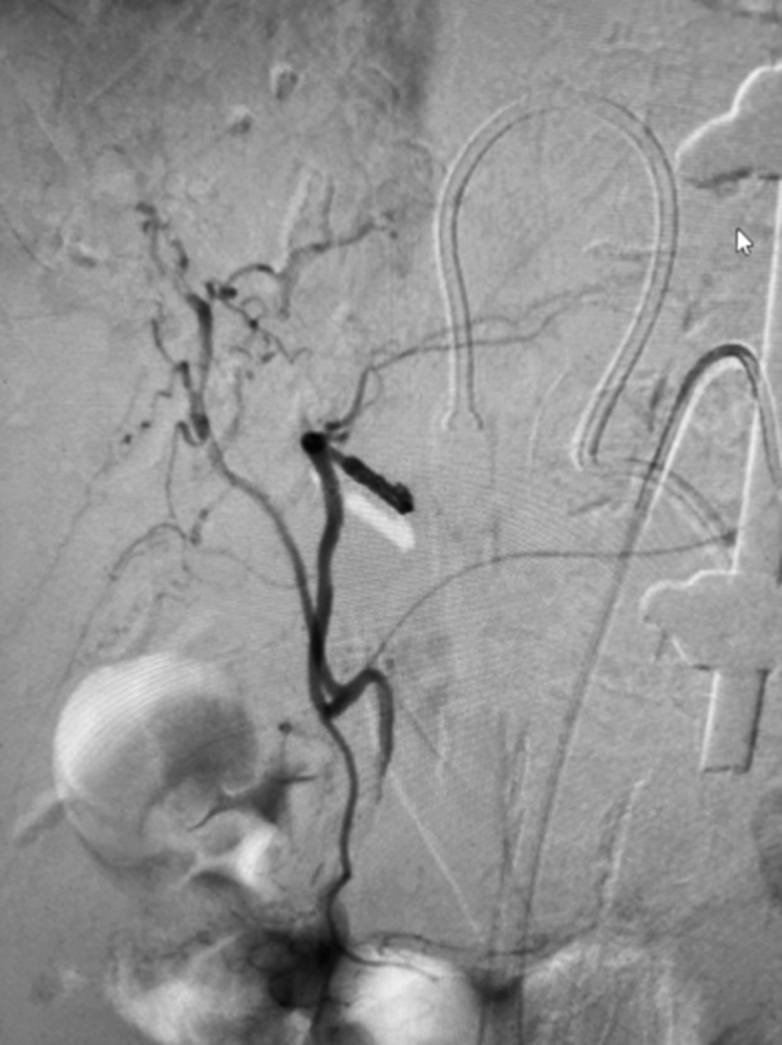
Angiogram Image showing pericardial drain course.

**Figure 9:**
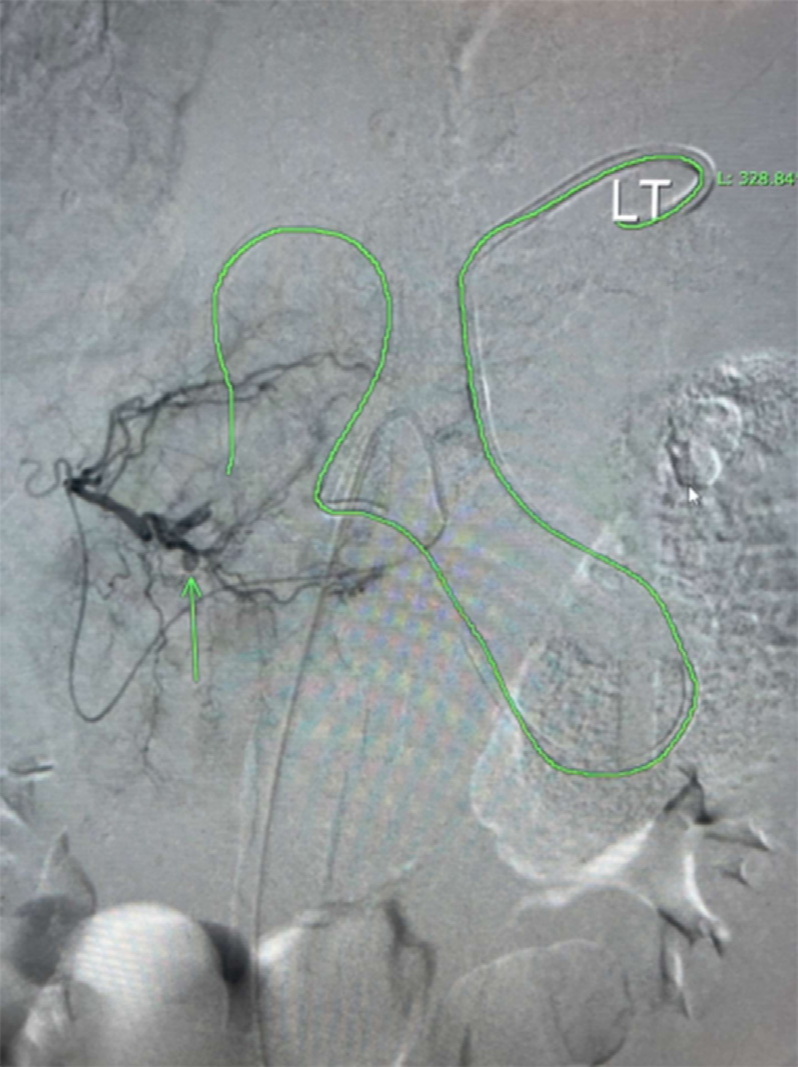
Postembolization Arteriography Image demonstrating complete occlusion of the pseudoaneurysm.

A massive transfusion protocol was initiated, and multiple vasopressors were started for a brief period. Left hepatic artery digital subtraction angiography (DSA) performed by the interventional radiology service demonstrated a pseudoaneurysm arising from segment III branch of the left hepatic artery (Figures 7 and 8). Coil embolization was performed with a 2 mm × 4 Penumbra Ruby soft coil and 5 cm packing coil (× 2). Postembolization arteriography demonstrated complete occlusion of the pseudoaneurysm (Figure 9). The patient received a transfusion of 4 units of packed red blood cells over the course. The patient was extubated on day 4 and was weaned off vasopressors on day 5. The pericardial drain had significant amounts of bloody output.

## Discussion

Pericardiocentesis, when performed with imaging guidance, is a relatively safe procedure, with the risk of complications ranging from 4% to 10%.^1^ The most common complications are arrhythmias, coronary artery or cardiac chamber puncture, hemothorax, pneumothorax, and pneumopericardium.^1^ We report a rare and novel case of hemoperitoneum due to hepatic laceration and hepatic artery pseudoaneurysm formation as a complication of emergent pericardiocentesis. To our knowledge, there has been no case report of liver laceration with hepatic artery pseudoaneurysm complicating pericardiocentesis.

The precise incidence of hemoperitoneum after pericardiocentesis is not known. Etiologies like injury to the left inferior phrenic artery have been described in case reports.^2^ The presentation can vary depending on the severity and nature of the cause; in our case it was gradual onset followed by acute decompensation.

Management depends on the underlying cause. Patients in stable condition can be treated conservatively, and those with hemodynamic instability may require surgical laparotomy. In our case, the patient was successfully treated with coil embolization of the hepatic artery pseudoaneurysm with guidance by the interventional radiology service.

## Follow-Up

**Figure 10:**
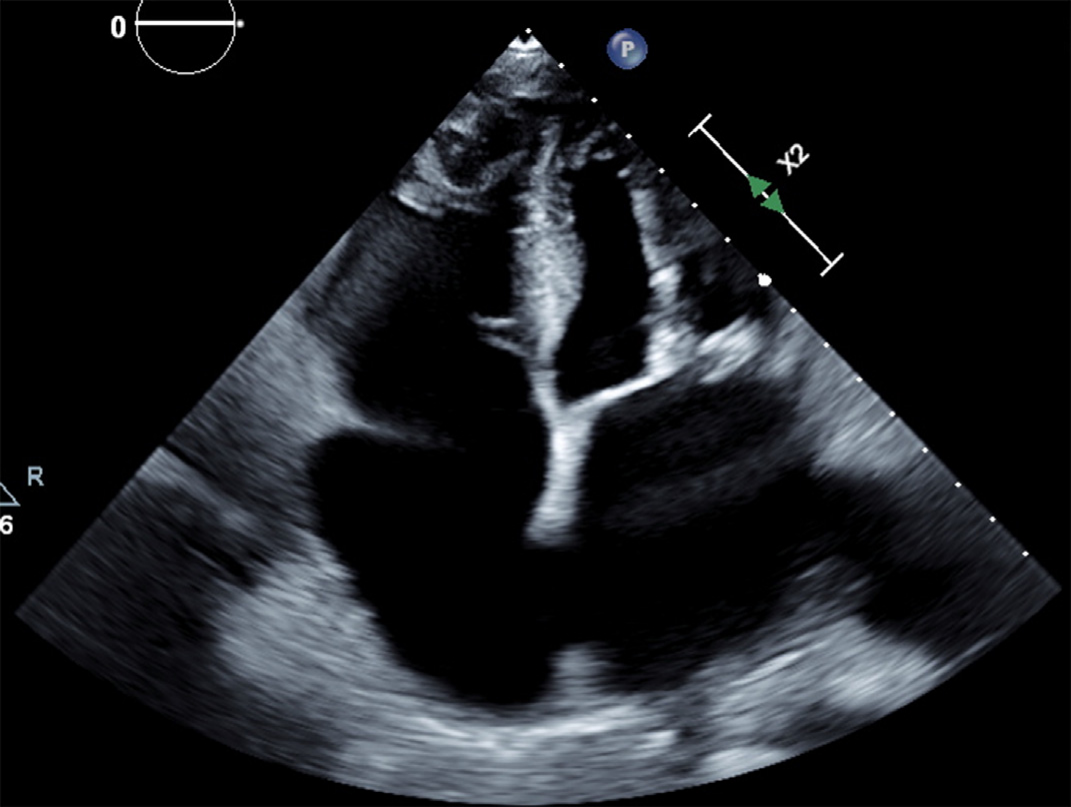
Transthoracic Echocardiogram Apical 4-chamber view demonstrating no pericardial effusion.

**Figure 11:**
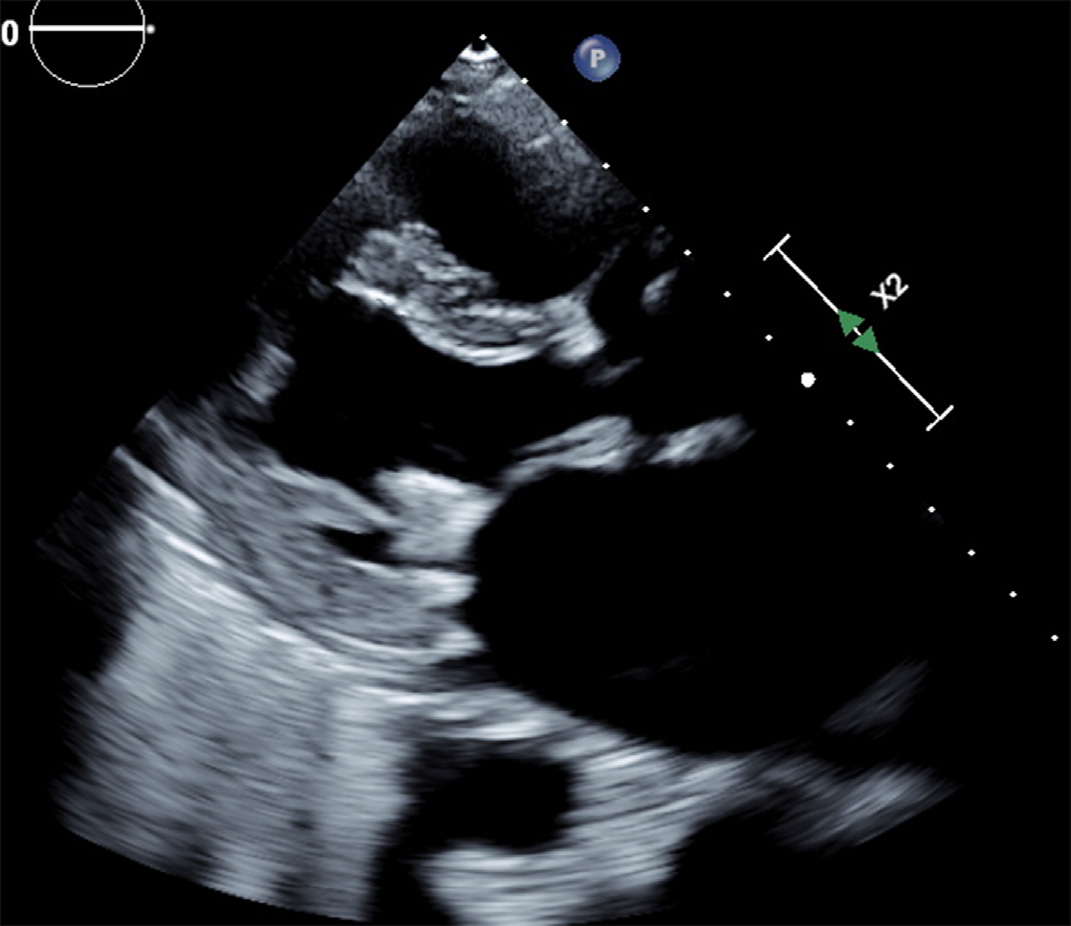
Transthoracic Echocardiogram Parasternal long-axis view demonstranting no pericadial effusion.

Because of the continued significant drain output, the pericardial drain was kept in situ with monitoring of drain output and TTEs. The pericardial drain was removed on postprocedure day 12. Follow-up TTE did not reveal any significant effusion (Figures 10 and 11, Supplemental Video 3, Supplemental Video 4, Supplemental Video 5).

The patient was planned for discharge to subacute rehabilitation, but she had a new-onset leukocytosis and complete opacification of the left lung. Intravenous antibiotic therapy was started because of possible pneumonia. Bronchoscopy revealed a large yellow-tan mucus plug down the left main bronchus, with corresponding left lung atelectasis. After 10 days of intravenous antibiotics and repeated bronchoscopy, the patient’s condition improved, and a postbronchoscopy chest x-ray showed significant improvement of the left hemithorax. The patient was discharged to subacute rehabilitation in stable condition after 35 days of hospitalization.

## Conclusions

Pericardiocentesis is a potentially life-saving procedure that carries a high risk of complications. In this regard, imaging support and careful planning of the proper entry site are fundamental for a safe and successful procedure.

## Funding Support and Author Disclosures

The authors have reported that they have no relationships relevant to the contents of this paper to disclose.

## Appendix

Supplemental Video 1 Transthoracic Echocardiogram. Subcostal view with moderate, predominantly anterior, pericardial effusion with pericardial tamponade.

Supplemental Video 2 Transthoracic Echocardiogram. Parasternal long-axis view showing resolution of pericardial effusion after pericardiocentesis and pigtail catheter placement.

Supplemental Video 3 Transthoracic Echocardiogram. Apical 4-chamber view demonstrating no pericardial effusion.

Supplemental Video 4 Transthoracic Echocardiogram. Parasternal long-axis view demonstratig no pericardial effusion.

Supplemental Video 5 Transthoracic Echocardiogram . Apical 4-chamber view demonstratig no pericardial effusion.
